# Association of fetuin A, adiponectin, interleukin 10 and total antioxidant capacity with IVF outcomes

**Published:** 2014-11

**Authors:** Mustafa Yen, Orkide Donma, Ferdag Yildizfer, Ozlem Ekmekci, Z Asli Karatas Kul, A Esat Imal, Zafer Keser, Emin Cagil, Murat Mengi, Hakan Ekmekci, Sezai Sahmay, Metin Donma

**Affiliations:** 1 *Cerrahpasa Medical Faculty, Istanbul University, Istanbul, Turkey.*; 2 *Ministry of Health, Diyarbakir Education and Research Hospital, Diyarbakir, Turkey.*; 3 *Faculty of Medicine, Namik Kemal University, Tekirdag, Turkey.*

**Keywords:** *Adiponectin*, *Interleukin 10*, *Fetuin A*, *Interleukin 1RA*, *Infertility*

## Abstract

**Background:**

Possible roles of anti-inflammatory factors as well as total antioxidative capacity in reproductive processes of women undergoing *in vitro* fertilization (IVF) are still being investigated and the contributions by some of them remain controversial.

**Objective:**

The aim of this study is to investigate the relationship between anti-inflammatory parameters and total antioxidative capacity (TAC) of the body during IVF. In this respect, adiponectin, interleukin-10 (IL-10), interleukin-1 receptor antagonist (IL-1RA), fetuin A and TAC analyses have been performed.

**Materials and Methods:**

In this prospective, non-randomized, controlled clinical study, sera obtained from 26 fertile (Group-1), and 26 infertile women before (Group-2) and after (Group-3) IVF treatment were analyzed. IL-1RA, IL-10, fetuin A, adiponectin and insulin were determined by ELISA. TAC was determined spectrophotometrically. Mann-Whitney U test, paired sample t-test, Wilcoxon signed-rank test as well as Pearson correlation analysis by SPSS were performed for statistical analysis.

**Results:**

Clinical pregnancy and live birth rates were determined as 30.8% and 23.1%, respectively, in pregnant group. For the pregnant, significant indirect correlations were detected between fetuin A and adiponectin (r=-0.843; p=0.035) as well as IL-10 (r=-0.846; p=0.034) in Group 2. The correlation between adiponectin and IL-10 doubled in pregnant compared to non-pregnant (r=0.929; p=0.007 vs. r=0.478; p=0.033). The correlations between fetuin A and TAC in pregnant were noted both in Group 2 (r=0.892; p=0.017) and Group 3 (r=0.875; p=0.022). No correlation of fetuin A with these parameters was detected in non-pregnant group.

**Conclusion:**

Fetuin A, TAC, IL-10, adiponectin and their associations may be important from their predictive values for IVF success point of view. Parameters with anti-inflammatory or antioxidant property appear to improve pregnancy in women undergoing IVF.

## Introduction

Studies on assisted reproduction techniques (ARTs) are developing to increase the success rates for achieving pregnancy (1). Currently, about 2-4% of the pregnancies are developed by in vitro fertilization (IVF). IVF can be defined as one of the ARTs medically applied on oocyte, sperm or embryo cells in vitro in order to develop pregnancy. Despite the recent advances to overcome the infertility-related problems, the success rates remain low. This leads to distress for the couples and is also important from the women’s health point of view (2, 3). Investigation of the factors affecting IVF outcome may improve the chances of a successful outcome (4-9). Reactive oxygen species may be involved in the pathogenesis of infertility and are suggested as the factors that negatively affect the outcomes of ARTs. Antioxidants may possibly reduce the negative effects of oxidative stress in female infertility. In this respect, it is expected that the determination of the antioxidative profile, oral antioxidant support or the enrichment of IVF milieu with antioxidants may contribute to the treatment strategies and may positively affect the growth of embryo (5-10). 

It is also important to integrate endocrine and cytokine systems and to detect whether anti-inflammatory factors as well as total antioxidative capacity (TAC) contribute to IVF success or not. The evaluation of some parameters, which contribute to anti-inflammatory system, mostly derived from adipose tissue along with TAC will handle the matter from another point of view. 

The recent studies have reported that antioxidant defense system may be related to IVF outcome (10). Currently limited evidence suggests that antioxidants improve fertility and trials have explored this area with varied results. There are reports suggesting that antioxidants are not associated with an increased live birth rate or clinical pregnancy rate (11). On the other hand, a higher overall antioxidant status in blood plasma was suggested as advantageous for achieving IVF pregnancy (12). In an experimental study, increasing oxidative stress impaired the development of oocytes and it has been shown that antioxidant supplementation by addition of antioxidants to IVF medium or maternal antioxidant injection overcomes the detrimental effects of stress-induced oxidative stress. It has been suggested that these data are helpful when making attempts to increase the chances of a successful outcome in women undergoing IVF intervention (5). 

It was found that the endometrial expressions of proinflammatory cytokines such as TNF-α were up-regulated and interleukin-10 (IL-10), as an important anti-inflammatory cytokine in pregnancy, was down-regulated in idiopathic recurrent spontaneous miscarriage women. The down-regulation of IL-10 is associated with impaired endometrial perfusion which possibly makes the endometrium unreceptive that may eventually cause early pregnancy loss (13). It is even reported that IL-10 levels between 14 and 18 weeks of gestation may act as potential early biomarkers in the diagnosis of preeclampsia (14). 

Fetuin A, a multifunctional protein, can be defined as the inhibitor of ectopic calcification in blood circulation (15, 16). Among adipokines and hepatokines, adiponectin and fetuin-A, regulate insulin sensitivity, are associated with insulin resistance and may be involved in development of diabetes (17-19). It is reported that fetuin A cannot be used as an estimate of fertilization success, because markers of reproductive potential appeared to be independent of fetuin A (16). 

Adiponectin, adipose tissue derived hormone and cytokine is known to have insulin-sensitizing, anti-inflammatory, and anti-atherogenic properties (20, 21). It influences gonadotropin release, pregnancy and assisted reproduction outcomes, acts to reduce insulin resistance (17, 22). Adiponectin has been shown to exert actions in female reproductive system and thus may influence fertility and pregnancy (20). Its receptors are present in many reproductive tissues such as ovaries, endometrium and placenta and adiponectin may be involved in mechanisms connecting reproduction and metabolism. Higher adiponectin levels are reported to be associated with better outcomes in assisted reproductive cycles (20, 22). Adiponectin can modulate follicle growth and also enhances oocyte maturation as well as early embryo development in mouse and human IVF procedures (23).

Interleukin-1 receptor antagonist (IL-1RA) is the natural antagonist of proinflammatory cytokine IL-1. It is suggested that it plays an important role in the regulation of inflammatory responses (24). Therefore it has an anti-inflammatory nature. Administration of IL-1RA significantly increased adiponectin levels in hemodialysis patients (21). The causes of infertility due to factors originated from women were considered in this study. The aim is to investigate the relationship between anti-inflammatory parameters and TAC during IVF. In this respect, the associations of adiponectin, IL-10, IL-1RA, fetuin A and TAC with IVF outcomes have been evaluated. 

## Materials and methods

In this prospective, non-randomized, controlled clinical study, the blood samples from 70 women, who consulted to the IVF Center, Obstetrics and Gynaecology Department, Cerrahpasa Medical Faculty, University of Istanbul between June 2009 and September 2010, with the complaint of infertility, and had the features of being between the ages of 23 and 40 years, being married at least for 3 years, having social security at least for 5 years and two times of intrauterine insemination before, were taken for analysis prior to the beginning of the treatment in order to determine the suitability for the participation into the study. 

A signed written informed consent was obtained from all participants prior to the study. Procedures were carried out in accordance with Declaration of Helsinki. Ethical approval to conduct the study was obtained from the Ethics Committee for the Clinical Investigations of Istanbul University, Cerrahpasa Medical Faculty. 

The medical history, routine gynecological exam, anthropometry, routine biochemical tests (complete blood cell count, fasting blood sugar, blood urea nitrogen, creatinine, alanine aminotransferase, aspartate aminotransferase, gamma glutamyl trans peptidase, blood group, Coombs test, urine analysis), ultrasonography, serology (HBsAg, AntiHBs, HCV, HIV, TORCH), basic infertility tests (spermiogram, hormonal tests, hysterosalpingography), and hormonal tests (AMH, inhibin B, FSH, LH, E_2_, prolactin, TSH) of the patients were performed to evaluate the causes of infertility before the treatment in order to enlighten the source of the problem.

Causes of reduced female fertility included decreased ovarian reserve, anovulation, uterine disorders other than endometriosis, polycystic ovary syndrome, and fertility-sparing surgery with unilateral salpingooophorectomy, methylene tetrahydrofolate reductase gene mutation, unexplained reasons and presence of more than one factor. The primary outcome was clinical pregnancy rate and the secondary outcome included live birth rate.

**Figure 1:**
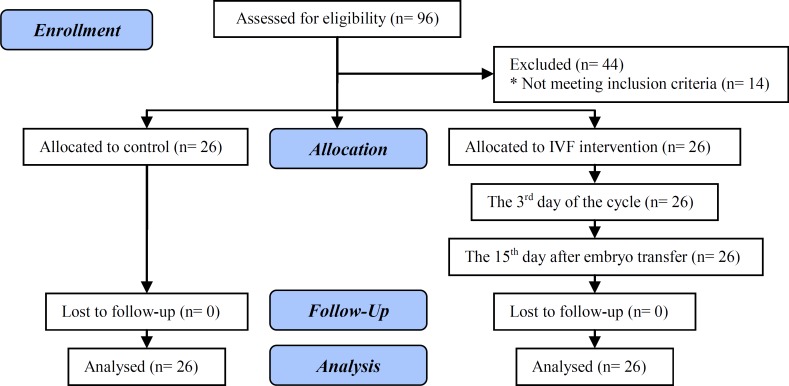
Flow diagram of participants through each stage of the study.

Twenty-six women, who had given birth without any medication constituted the control group (Group 1). The blood samples were drawn from 70 infertile women during the early follicular phase (the 3^rd^ day of the cycle) before the onset of the IVF intervention. In total 26 managed to complete the process with the appropriate respond to treatment. Their “pre-IVF samples” (Group 2) out of 70 were included into the study to constitute the paired data with the “post-IVF samples” (Group 3) taken on the 15th day of the application of embryo transfer from these 26 women. Figure 1 shows the flow diagram of participants through each stage of prospective, non-randomized, controlled clinical study. 

Anthropometric measurements and demographic characteristics of the women participated in the study were recorded. A total of 122 blood samples including the control group samples were taken into sterile vacuum operated tubes at 0800-1000 am while fasting before the IVF treatment on the 3^rd^ day of the menstruation (follicular phase) and on the 15^th^ day after the embryo transfer. The samples were centrifuged in 2000 rpm for 10 min. The sera were removed and stored at -80ºC until assayed. 

Adiponectin, IL-10, IL-1RA, insulin, fetuin A values of the samples were determined by enzyme-linked immunosorbent assay (ELISA). TAC levels were determined spectrophotometrically. All samples were assayed using the RayBio Human IL-1ra ELISA kit (RayBiotech, Inc, Norcross, GA, USA), Insulin ELISA kit (DRG Instruments GmbH, Germany), the AssayMax Human Adiponectin ELISA kit (AssayPro, St. Charles, MO, USA), the AssayMax Human IL-10 ELISA kit (AssayPro, St. Charles, MO, USA), the AssayMax Human alpha-2-HS-Glycoprotein (AHSG) ELISA kit (AssayPro, St. Charles, MO, USA) and the Antioxidant Assay kit (Cayman Chemical Company, Ann Arbor, MI, USA). 

**Statistical analysis**

Statistical evaluations were performed by the Statistical Package for Social Sciences (SPSS) Version 13.0 software package. Data were analyzed using descriptive-analytic tests. Parametric variables were represented as mean and standard error (SE), and categorical data were represented by number (n) and percentage (%). Simple distribution of the study variables and cross tabulation was applied. The values for arithmetical mean, SE were calculated for the pregnant and non-pregnant women in pre-IVF and post-IVF groups as well as for the participants in control group. Mann Whitney U test, paired sample t-test, Wilcoxon signed-rank test as well as correlation analysis were used. Correlation coefficients (r) and the exact p values were calculated. The results in all the above mentioned procedures were accepted as statistically significant when the p-value was less than 5% (p<0.05).

## Results

In IVF group, the mean age and body mass index (BMI) were calculated as 31.2±1.4 years and 25.6±2.8 kg/m^2^, respectively. The corresponding values in the control group were 31.4±1.5 years and 24.1±1.1 kg/m^2^. There was no statistically significant difference between the ages and BMI values of the control and the patient groups. Out of 26 women, who were included into the scope of the study, 8 developed pregnancy after IVF. Since 2 underwent medical abortion due to empty sac (embryonic pregnancy), 6 concluded with live birth. 

Therefore, clinical pregnancy rate and IVF success were determined as 30.8% and 23.1%, respectively. The mean±SE and the exact p values for adiponectin, IL-10, IL-1RA, insulin, fetuin A and TAC of the control (G1) as well as pre- (G2) and post- (G3) IVF samples were tabulated in Table I. No correlation was detected in control group. However, significant correlations were observed between fetuin A and adiponectin (r=-0.531; p=0.005) as well as TAC (r=0.412; p=0.036) in pre-IVF samples (p≤0.05). There was also significant correlation between adiponectin and IL-10 (r=0.648; p=0.001). Statistically significant correlations were observed between IL-10 and insulin (r=0.448; p=0.022), adiponectin and IL-1RA (r=-0.693; p=0.001) as well as TAC (r=0.443; p=0.023) after IVF. 

It was also important to note the significant associations in IL-10 levels (r=0.436; p=0.026) as well as IL-1RA levels (r=-0.424; p=0.031) between pre- and post-IVF values. For the pregnant in the pre-IVF group, important correlations were detected between fetuin A and adiponectin (r=-0.843; p=0.035), IL-10 (r=-0.846; p=0.034) as well as TAC (r=0.892; p=0.017). A significant correlation between adiponectin and IL-10 (r=0.929; p=0.007) was also noted in this group. There was also an association between fetuin A and TAC (r=0.875; p=0.022) in post-IVF values of this group. No correlation of fetuin A with these parameters was detected in non-pregnant group.

**Table I:** The mean ± SE and p values for adiponectin, IL-10, IL-1RA, insulin, fetuin A and TAC of the control (G1) as well as pre- (G2) and post- (G3) IVF samples

**Parameter**	**Control (G1)**	**Pre-IVF (G2)**	**Post-IVF (G3)**	**p-value**	
Adiponectin (μg/ml)	6.6±0.6	11.4±0.5	8.5±0.5	G1 vs. G2	0.001
				G1 vs. G3	0.013
				G2 vs. G3	0.001
IL-10 (pg/ml)	260±108	240±10	190±6	G1 vs. G2	0.848
				G1 vs. G3	0.556
				G2 vs. G3	0.001
IL-1RA (pg/ml)	410 ± 59	860 ± 71	670±73	G1 vs. G2	0.001
				G1 vs. G3	0.008
				G2 vs. G3	0.133
Insulin (μIU/ml)	14.3±2.7	8.4±1.0	15.6±3.1	G1 vs. G2	0.045
				G1 vs. G3	0.757
				G2 vs. G3	0.019
Fetuin A (μg/ml)	889±75	825±39	921±56	G1 vs. G2	0.460
				G1 vs. G3	0.732
				G2 vs. G3	0.105
TAC (mM Trolox)	1.0±0.1	1.0±0.1	1.2±0.1	G1 vs. G2	0.933
				G1 vs. G3	0.026
				G2 vs. G3	0.014

Data are presented as mean ±SE.

IL: interleukin

RA: receptor antagonist

TAC: total antioxidative capacity

## Discussion

In this study, the aim was to gather some information on some anti-inflammatory parameter profiles as well as TAC as the indicator of total amount of antioxidants and also to enlighten the association of them with IVF process. There are various reports on the rates of success in IVF trials. Clinical pregnancy rates were reported as 43.4% in the United States and 29.7% in Europe. The corresponding values of live birth rates were declared as 38.2% and 27.6% (25). A study from Turkey has reported clinical pregnancy rate as 30% in 2010 (26). In the current study, clinical pregnancy and live birth rates after IVF were found as 30.8% and 23.1%, respectively. 

Studies on antioxidants during IVF intervention have gained importance in recent years. Antioxidants may reduce the negative effects of oxidative stress in female infertility and may contribute to the treatment strategies and positively affect the growth of embryo. Supplementation of antioxidants to IVF medium or injection of antioxidants into mice models significantly improved the development of blastocytes and oocytes, which contribute to increased IVF success (5). It is reported that in vitro ascorbic acid supplementation could protect teratozoospermic specimens against oxidative stress and it could improve ART outcome (27). In a recent study, women who became pregnant presented higher plasma vitamin E concentrations than those who did not (10). In another study, higher systemic blood total antioxidant response was significantly favorable for achieving clinical pregnancy (12).

In our study, as IVF intervention caused an increase in TAC proceeding from pre-IVF towards post-IVF (p=0.014), significant difference was observed between pregnant and non-pregnant neither in pre-IVF nor post-IVF samples. However, in pregnant group, fetuin A-TAC association observed both in pre-IVF and post-IVF samples was remarkable. Upon examination of the values obtained before IVF trial, higher values were detected in insulin, fetuin A and TAC in the group developing pregnancy when compared to the values in non-pregnant group. Higher adiponectin, IL-10, fetuin A values were noted following IVF in women, who gave birth.

Fetuin A is defined as the inhibitor of ectopic calcification in circulation (28, 29). This parameter has already been measured in patients with chronic renal diseases, coronary arterial diseases, Helicobacter pylori infection, and those undergoing haemodialysis (28-32). In pregnant women fetuin A levels were shown to increase with gestational age (33). Fetuin A plays role in counter-regulating injury- or infection-elicited inflammatory responses (34). Administration of fetuin A may be beneficial to protect against lethal systemic inflammation (35). Reduced fetuin A levels in trisomy 21 may potentially be associated with growth restriction or impaired osteogenesis (36). Its decreased concentration may reflect systemic inflammation in preeclampsia (37). Defects and impaired function of fetuin A in fetuses with intrauterine growth restriction may be responsible for deficient fetal growth as well as osteogenesis (38). It has been reported that serum fetuin A levels of patients participating IVF are markedly elevated when compared to those of healthy women, however it cannot be used as an estimation of fertilization success (16). However, the authors did not investigate the association of this parameter with adiponectin or TAC. We have found fetuin A-TAC association in both pre-IVF and post IVF samples of the pregnant group.

Serum fetuin A levels were reported to be negatively correlated with serum adiponectin in diabetes (19). A negative correlation between fetuin A and adiponectin has also been shown in general population studies (39). There are some reports pointing out the association of the adiponectin and fetuin A independently of each other with some disease states (18). In our study, we have reported that the association of these two parameters in pre-IVF samples of the pregnants may be used as the indicator of IVF success. We have also detected some other meaningful correlations of an antioxidative cytokine, IL-10, both with fetuin A and adiponectin in the same group. 

In our study, the difference between pre- and post-IVF fetuin A levels in the pregnant group, was much higher than that in the non-pregnant group. These findings may suggest that the increase in fetuin A levels may be directly proportional to the IVF success. While there were no correlation between the parameters in the control group, the presence of significant correlations between fetuin A and adiponectin as well as TAC in pre-IVF group have pointed out the necessity of a more detailed investigation of the profile related to the matter. In this respect, in pre-IVF samples obtained from the women who developed pregnancy, significant correlations between fetuin A and adiponectin, IL-10 as well as TAC were found. The absence of correlation between fetuin A and these parameters in non-pregnant group made these relations detected in pregnant group more remarkable. 

The association between fetuin A and TAC in pregnants following IVF was unique. In pre-IVF samples, this was the only correlation, which is also ongoing in post-IVF samples (r=0.892; p≤0.017 vs. r=0.875; p≤0.022). The degree of correlation between adiponectin and IL-10 before IVF was almost twice in pregnant (r=0.929; p≤0.007) in comparison with that of non-pregnant (r=0.478; p≤0.033).

## Conclusion

In conclusion, fetuin A, TAC, IL-10, adiponectin and their associations may be important from their predictive values for IVF success point of view. Particularly, the relationship between fetuin A and TAC during IVF may be important to achieve live birth. Parameters with anti-inflammatory or antioxidant property appear to improve pregnancy in women undergoing IVF.

## Note

***This article extracted from M.Sc. thesis. (Mustafa Yen)***
